# Pozzolanic activity experimental dataset of calcined coal gangue

**DOI:** 10.1016/j.dib.2023.109802

**Published:** 2023-11-14

**Authors:** Junfei Zhang, Haoyang Han, Ling Wang

**Affiliations:** School of Civil and Transportation Engineering, Hebei University of Technology, Tianjin, 300401, China

**Keywords:** Calcination scheme, Coal gangue, Pozzolanic activity, Strength comparison method, R3 activity test method

## Abstract

The coal gangue in this dataset was subjected to a series of processes, including drying, crushing, and milling. Subsequently, the coal gangue powder was subjected to high-temperature calcination in a muffle furnace, with a heating rate of 4 ℃/min. The pozzolanic activity of coal gangue powder was investigated at various calcination temperatures (600 ℃, 700 ℃, 800 ℃, 900 ℃) and different holding times (1h, 2h). Cement mortar specimens containing calcined coal gangue powder were prepared, and their compressive and flexural strengths were tested to evaluate the reactivity of the calcined coal gangue. In addition, the Rapid, Relevant and Reliable (R3) activity test was conducted to test the reactivity. The thermogravimetric analyzer was employed to determine the TG-DTG curves of coal gangue powder. X-ray diffractometer, Fourier infrared spectrometer and scanning electron microscope were utilized to investigate the microstructure of activated coal gangue powder at different temperature ranges. These data can be used for determining the optimal calcination scheme of coal gangue to maximize its potential as a partial cement clinker replacement in cement production, thereby contributing to cost reduction and carbon emission mitigation.

Specifications Table

**Table:** 

Subject area	Engineering, Material Science, Cements and Concrete
Specific subject area	Solid Waste Materials, Pozzolanic Cements and Blends, Cement Replacements and Sustainable Materials
Data format	Raw and analyzed
Type of data	Table, graphs and figure
Data collection	Data were collected from coal gangue powder subjected to different calcination temperatures and holding times. Details are described in this article. The compressive and flexural strength data of resin sand test specimens were obtained using the YAW-300 electro-hydraulic servo pressure testing machine. The R3 reactivity test results combined with water loss were obtained using a muffle furnace (0-1000 ℃) and a high-precision electronic balance (0.01g). Additionally, we collected thermogravimetric-differential thermal analysis curves, microstructural morphology and compositional data of coal gangue powder.
Data source location	The tests was conducted at the School of Civil Engineering and Transportation Engineering, Hebei University of Technology, located in Tianjin, China.
Data accessibility	Repository name: Pozzolanic-activity-experimental-dataset-of-calcined-coal-gangue-v1.0.2 [1] Data identification number/DOI:10.5281/zenodo.10049352 Direct URL to data: https://zenodo.org/records/10049352

## 1 Value of the Data

• These data will contribute to the widespread application of coal gangue calcination systems in research, stimulating the pozzolanic activity of coal gangue to maximize its utilization as a partial replacement for clinker in cement production processes, thereby reducing costs and carbon emissions;
• These data provide information on the microstructure and phase composition of coal gangue powder before and after calcination;
• These data will be beneficial for other scholars to extensively apply the calcination schemes to produce high-activity coal gangue powder, aiming to promote the resource utilization and value enhancement of this solid waste material [2];
• The experimental data are valuable for engineers and manufacturers. They can refer to the coal gangue calcination regimes obtained in this study to seek more suitable regimes for implementation in manufacturing processes. Furthermore, these findings can also be applicable to similar solid waste materials, aiding in the exploration of sustainable alternatives for cement.

## 2 Data Description

The dataset encompasses the reactivity of raw coal gangue under various calcination schemes. The coal gangue is subjected to a process of drying, crushing, and grinding, followed by high-temperature calcination using a muffle furnace with a heating rate of 4 ℃/min. The effects of calcination temperature (600 ℃, 700 ℃, 800 ℃, 900 ℃) and holding time (1h, 2h) on the pozzolanic activity of coal gangue powder are investigated, and the reactivity indices are determined using the strength comparison method and R3 reactivity test method. Microscopic characterizations including thermogravimetric testing, X-ray diffraction (XRD), Fourier transform infrared spectroscopy (FTIR), and scanning electron microscopy (SEM) are conducted on the calcined coal gangue powder to obtain data on the microstructure and phase composition of activated coal gangue powder.

**Fig. 1:**
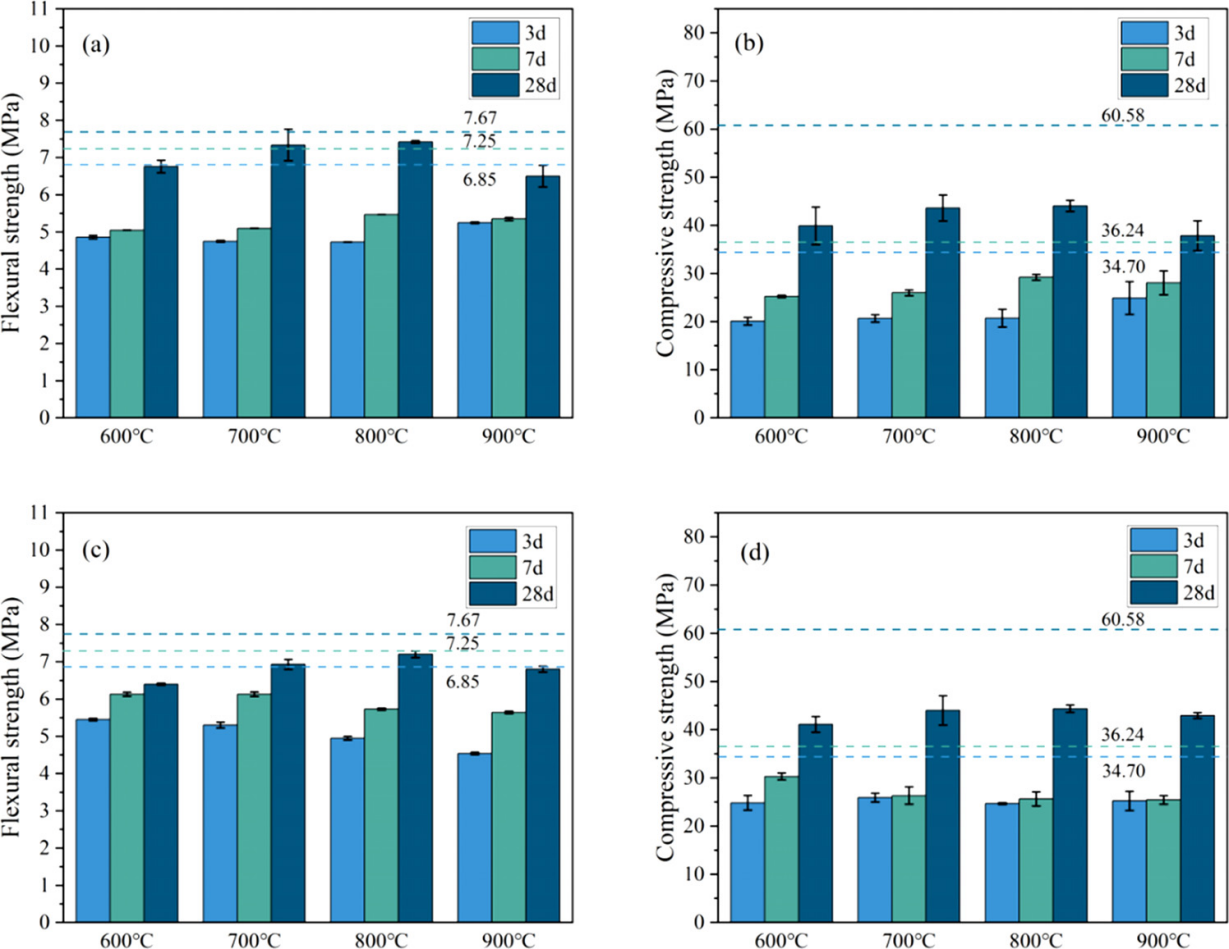
Flexural and compressive strength of calcined coal gangue concrete for different calcination times: (a&b) 1 h, (c&d) 2h.

**Fig. 2:**
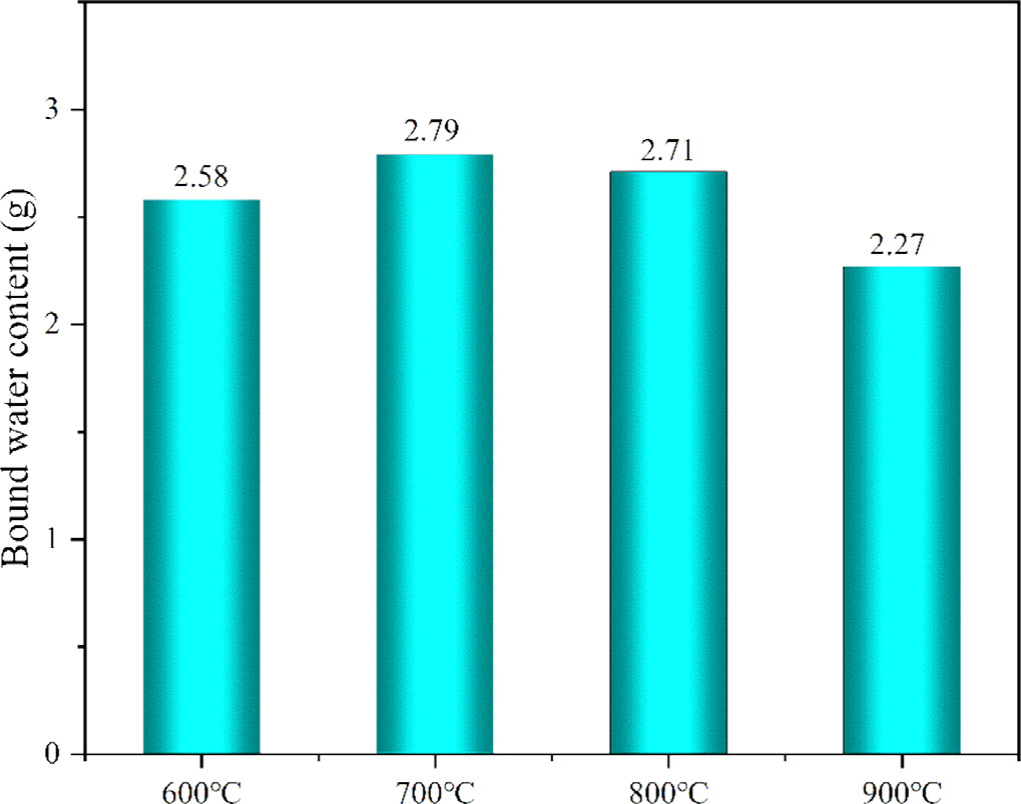
R3 Bound water content.

**Fig. 3:**
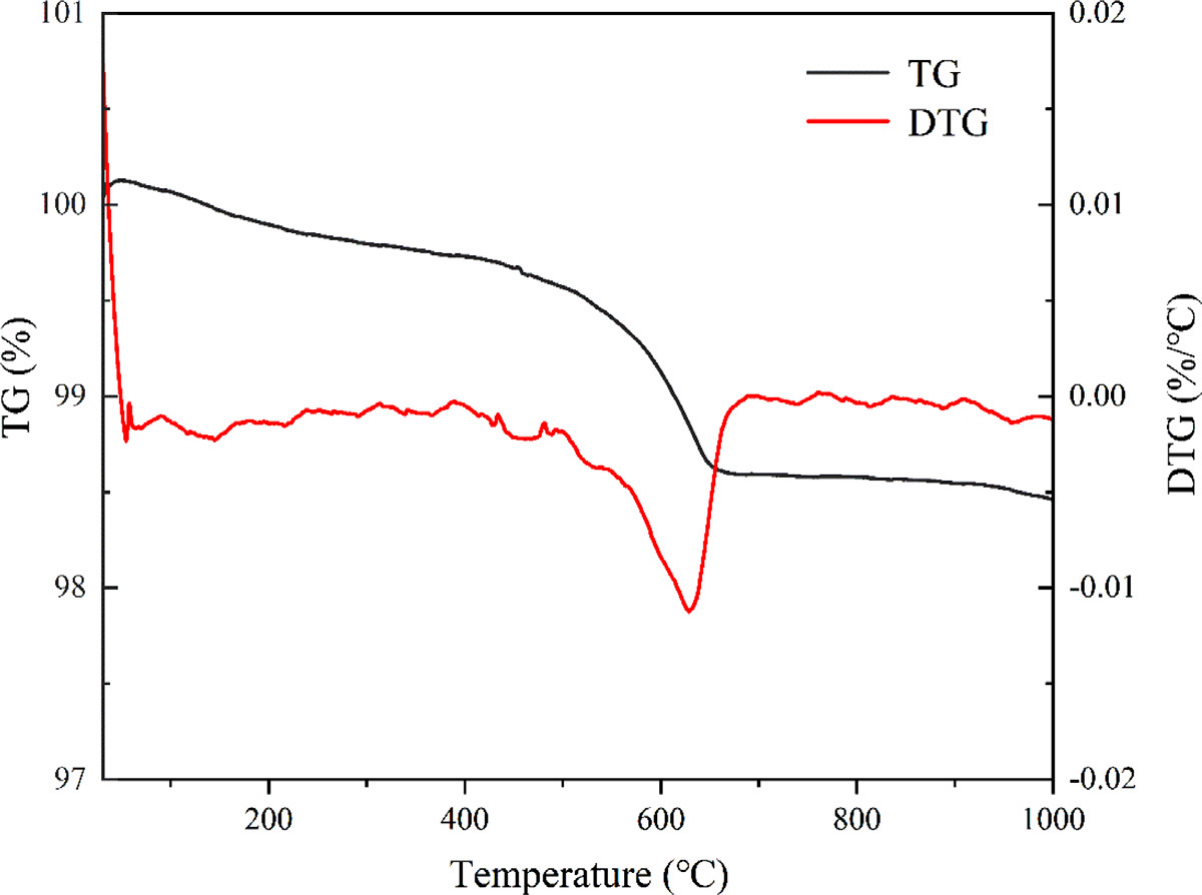
TG-DTG curves of raw coal gangue.

**Fig. 4:**
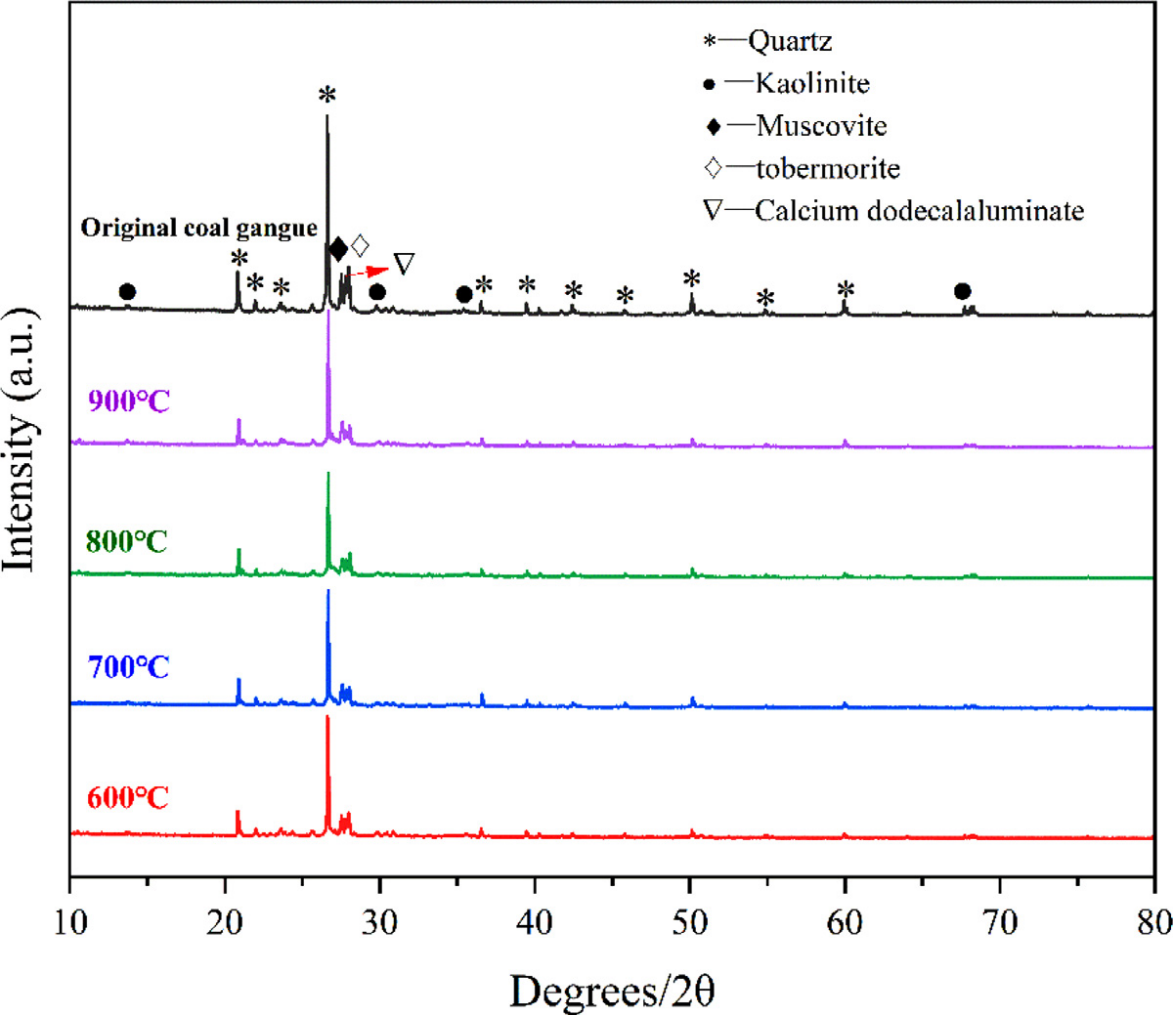
XRD of coal gangue at different temperatures.

**Fig. 5:**
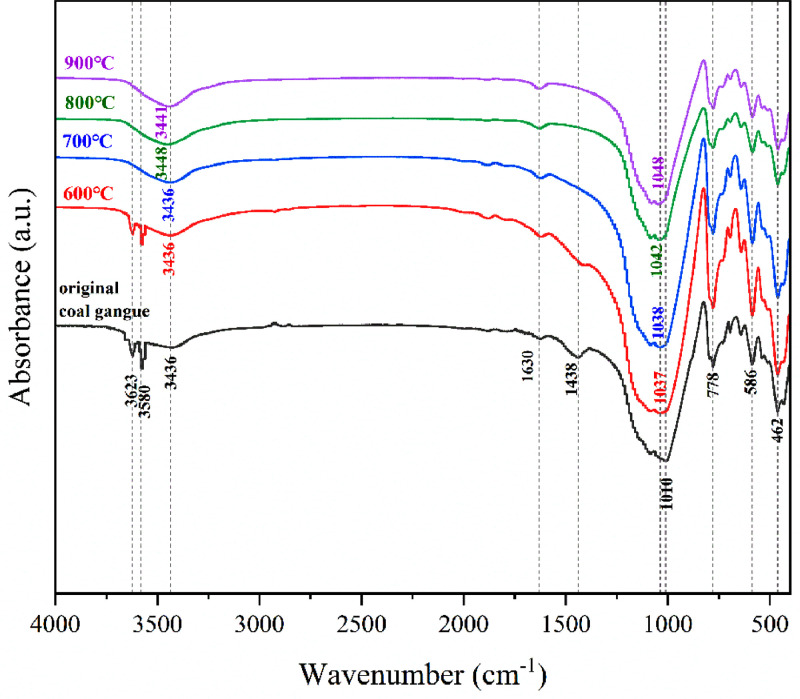
FTIR of coal gangue at different temperatures.

**Fig. 6:**
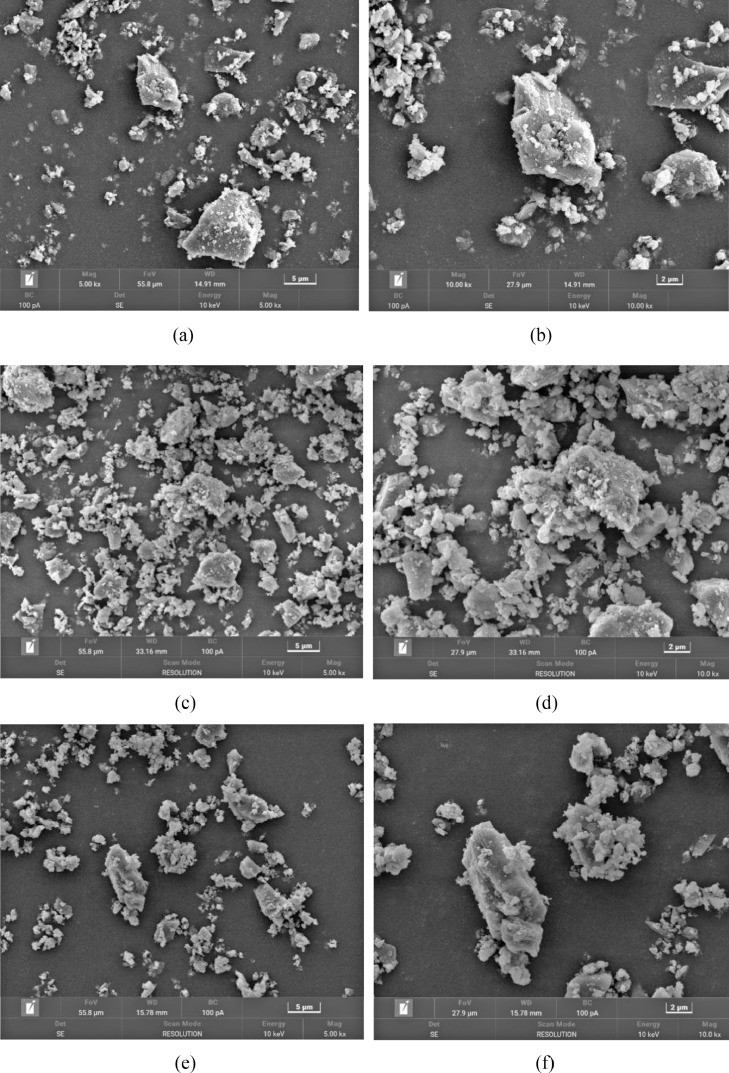

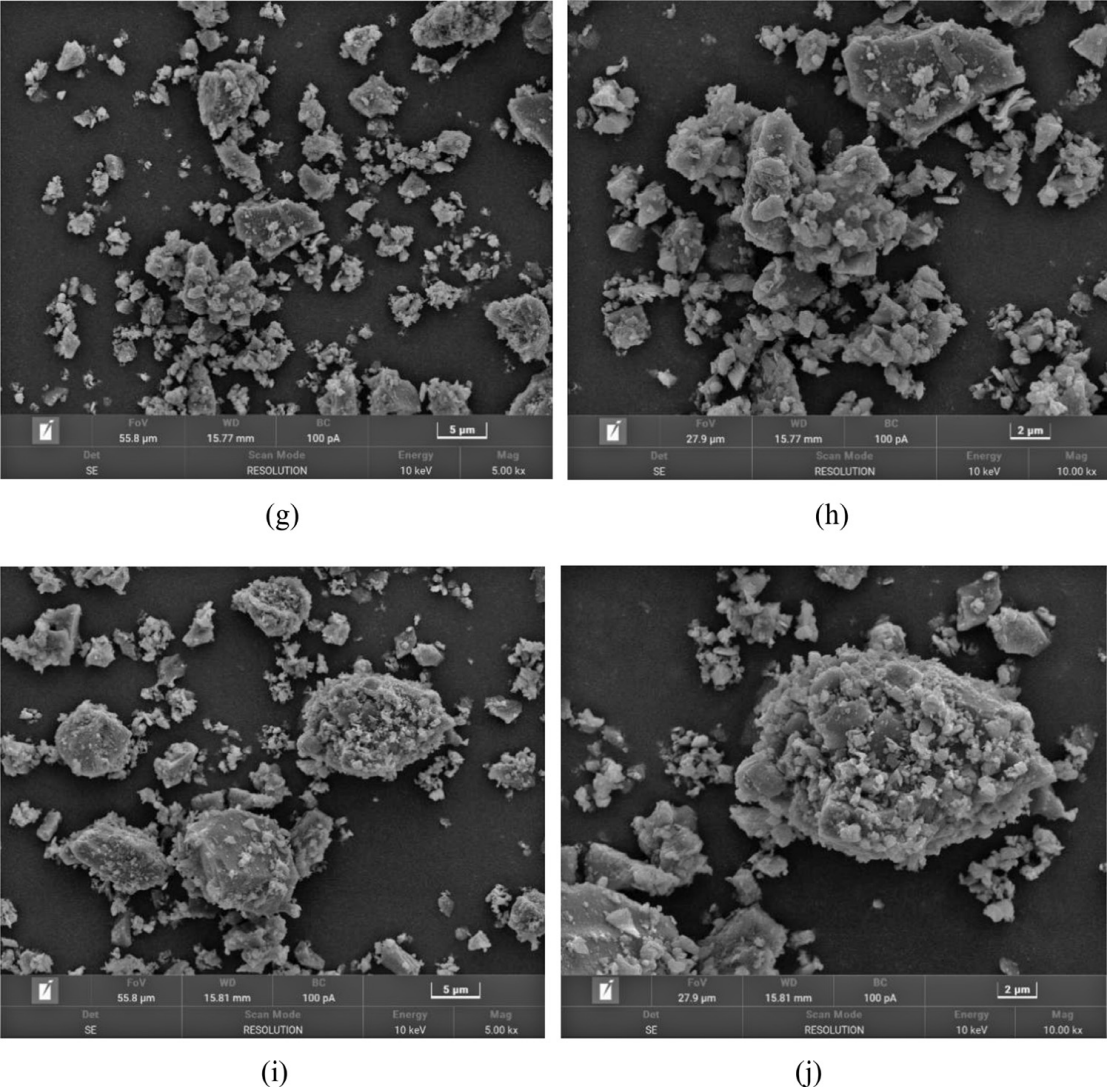
Micro-structure of calcined coal gangue: raw coal gangue (a and b), 600 °C (c and d), 700 °C (e and f), 800 °C (g and h), and 900 °C (i and j).

Table 1 shows the flexural and compressive strength of specimens containing coal gangue powder from different temperature after curing for 3, 7, and 28 days. Fig. 1 illustrates the flexural and compressive strength of the specimens. Fig. 2 shows R3 Bound water content. Fig. 3 exhibits the TG-DTG curves of the original coal gangue powder. Fig. 4 displays the XRD curves of coal gangue powder from different temperature. Fig. 5 presents the FTIR curves of coal gangue powder from different temperature. Fig. 6 shows the microstructure of coal gangue powder from different temperatures. All detailed data are documented in the dataset (https://zenodo.org/records/10049352). “The data on the strength” presents the compressive and flexural strength data of cement mortar specimens (40 × 40 × 160 cm) containing 30 % calcined coal gangue at different temperatures and curing times (3 days, 7 days, and 28 days). “Column chart of strength” visually represents the flexural and compressive strength data mentioned above in the form of bar charts, with temperature intervals on the x-axis and flexural strength and compressive strength on the y-axis. “R3 activity test data” displays the weights before and after calcination, along with the weight difference representing the combined water content measured through R3 activity testing. “The bar chart of R3 activity test” visually represents the combined water content in the form of bar charts, with temperature intervals on the x-axis and combined water content on the y-axis. Thermogravimetric data show the changes in TG and DTG concerning temperature(T). FTIR curve data at different temperatures include Wavenumber and absorbance values. XRD curve data display Degrees and Intensity, along with 80 scanning electron microscope images capturing different temperature coal gangue powder photos.

**Table 1:** Strength of pulverized coal gangue mortar.

Group	Flexural strength (MPa)			Compressive strength (MPa)		
	3d	7d	28d	3d	7d	28d
Control group	6.85	7.25	7.67	34.70	36.24	60.58
600 ℃/1h	4.86	5.05	6.76	20.10	25.25	39.90
700 ℃/1h	4.82	5.10	7.34	19.91	26.00	43.62
800 ℃/1h	4.73	5.47	7.42	20.71	29.22	44.03
900 ℃/1h	5.25	5.35	6.50	24.92	28.57	37.85
600 ℃/2h	5.45	6.13	6.40	24.84	30.30	41.12
700 ℃/2h	5.30	6.13	6.93	25.93	26.33	44.00
800 ℃/2h	4.95	5.73	7.48	24.68	25.65	44.32
900 ℃/2h	4.54	5.64	6.80	25.23	25.43	42.93

## 3 Experimental Design, Materials and Methods

### 3.1 Experimental design

The raw coal gangue was dried using a blast drying machine (100 ℃, 2h), then crushed and ground using a tungsten carbide jaw crusher and a tungsten carbide disc mill (with an 80μm square sieve and less than 10 % residual). The coal gangue powder was subjected to high-temperature calcination using a muffle furnace with a heating rate of 4 ℃/min. Obtaining the pozzolanic activity of coal gangue powder at different calcination temperatures (600 ℃, 700 ℃, 800 ℃, 900 ℃) and holding times (1h, 2h) . The coal gangue powder from each temperature of calcination was substituted for cement clinker at a proportion of 30 %, and the compressive and flexural strengths were tested according to the strength comparison method [3]. The pozzolanic activity of calcined coal gangue was also evaluated using the R3 reactivity test method [4]. The microstructure and phase composition data of the raw coal gangue and the coal gangue calcined at different temperatures were derived.

### 3.2 Materials

The coal gangue used in this experiment was sourced from Shandong Huafeng Coal Mine. It predominantly exhibits a blackish-gray appearance with a predominantly sheet-like or block-like shape, displaying variations in size and shape. After drying and grinding the coal gangue, X-ray fluorescence (XRF) testing was conducted, and the results are presented in Table 2. According to the Chinese National Standard GB/T 35986-2018 [5], the LOI (loss on ignition) of the coal gangue powder was determined, which was found to be 13.85 %.

**Table 2:** Chemical composition of coal gangue.

Chemical composition	SiO_2_	Al_2_O_3_	Fe_2_O_3_	CaO	MgO	Na_2_O	K_2_O
Mass fraction ( %)	52.03	20.20	8.74	3.23	4.97	4.35	5.19

The cement clinker used in this study was Type I Portland cement with a grade of 52.5. X-ray fluorescence (XRF) testing was conducted on the cement clinker, and the elemental composition is presented in Table 3. The physical properties of the cement are provided in Table 4. The sand used in the study complied with GB/T 17671-2021 [6]. It consists of naturally occurring rounded silica sand with a SiO2 content not less than 98 %, and its particle size ranges from 0.08 to 2mm.

**Table 3:** Chemical composition of cement.

Chemical composition	CaO	SiO_2_	Al_2_O_3_	Fe_2_O_3_	SO_3_	MgO	K_2_O
Mass fraction (%)	69.58	16.42	4.77	3.10	2.64	1.72	0.60

**Table 4:** Physical properties of cement.

Materials	Stability	Setting time (min)		Compressive strength (MPa)		Flexural strength (MPa)	
		Initial	Final	3d	28d	3d	28d
PI-52.5 grade cement	Qualified	128	179	31.7	59.9	5.7	8.6

### 3.3 Methods

#### 3.3.1 Strength comparison method

According to the strength comparison method, cement mortar specimens were prepared by substituting 30 % of cement clinker with coal gangue powder. A control experiment without coal gangue powder was also conducted. The mortar specimens were prepared following the requirements of GB/T 17671-2021 [6], with a cement to standard sand ratio of 1:3 and a water-to-cement ratio of 0.50. After mixing in a planetary mixer, the mortar was molded on a vibration table to form standard prismatic test specimens (40 × 40 × 160mm). The specimens were then cured in a standard curing chamber at a temperature of 20 ± 1 ℃ and a humidity of not less than 90 % for 3 days, 7 days, and 28 days, respectively. The compressive and flexural strengths of the resin sand specimens were then tested using the YAW-300 electro-hydraulic servo pressure testing machine. The pozzolanic activity index of the coal gangue powder was calculated by comparing the strengths of the cement mortar with coal gangue powder to those of the same age control group without coal gangue powder.

#### 3.3.2 R3 activity test method

**Fig. 7:**
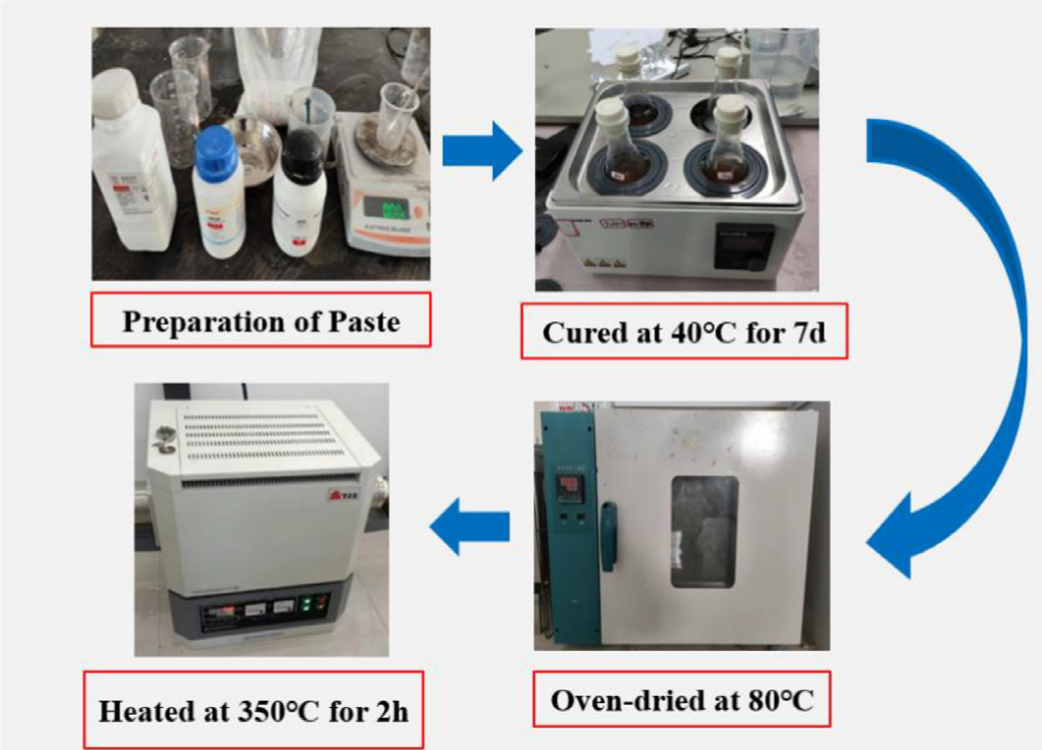
R3 activity test.

As shown in Fig. 7, the coal gangue reactivity was evaluated using the R3 test method according to the proportions specified in Table 5. The coal gangue from each temperature was used to prepare the paste, which was then sealed and cured in a water bath at 40 °C for 7 days. Subsequently, the cured samples were sliced and dried in an oven at 80°C until a constant weight was achieved. The dried samples were then heated in a furnace at 350 °C for 2 h, and the loss of combined water was calculated based on the difference in weight before and after calcination. The weight difference represents the loss of combined water.

**Table 5:** Mixture ratio.

Materials	CCG	Ca(OH)_2_	LS	KOH	K_2_SO_4_	Water
Weight (g)	11.11	33.33	5.56	0.28	1.31	60

#### 3.3.3 Thermogravimetric analysis

The thermalgravimetric analyzer (TGA) manufactured by Mettler in Switzerland was used to study the relationship between the mass of coal gangue powder and the temperature. Approximately 10 mg of powder sample was accurately weighed and placed inside an alumina crucible, which was then positioned on the microbalance of the TGA instrument. An empty alumina crucible was placed on the other side as a reference. After securing the sample, the instrument was started, and the temperature was increased from room temperature to 1000 °C at a heating rate of 10 °C/min. Nitrogen gas was used as the testing atmosphere, and TG-DTG curves were generated.

#### 3.3.4 X-ray diffraction test

The coal gangue powder was subjected to X-ray diffraction (XRD) testing using a SmartLab SE X-ray diffractometer manufactured by Rigaku, Japan. The sample does not contain any magnetic elements, and its phase and crystal structure were observed. A suitable amount of well-prepared powder sample was placed in the grooves of glass slides that were wiped with alcohol and then smoothed. The glass slides were placed on the sample stage of the diffractometer, and a copper target was used for the scan. The scanning range was set between 10 to 80 degrees, with a scanning speed of 5°/min. The data were processed using JADE 6.5 software.

#### 3.3.5 Fourier-transform infrared test

The coal gangue powder was subjected to Fourier-transform infrared (FTIR) spectroscopy using a Nicolet iS 10 instrument manufactured by Thermo Fisher Scientific, USA. The FTIR analysis involved inferring the phase composition and conducting phase transition analysis based on the molecular structure interruptions. The KBr pellet method was used as the testing technique, with a mass ratio of approximately 100:1 between the spectrally pure potassium bromide (KBr) and the powder sample. The FTIR spectra were obtained with a resolution of 0.4 cm-1 and an average of 128 scans.

#### 3.3.6 Scanning electron microscope test

The microstructure of coal gangue powder was captured using a scanning electron microscope (SEM) model S4800, manufactured by Hitachi, Japan. The samples were directly adhered to conductive adhesive and coated with a thin layer of gold without performing backscattering. The observation included both morphology and energy-dispersive X-ray spectroscopy (EDS) point scanning, with an energy range for EDS analysis spanning from Be4 to U92. The prepared samples were placed inside the sample chamber of the scanning electron microscope, and the lens distance and magnification were adjusted to initiate observation and capture images. The instrument offers a maximum resolution of 1.4 nm, magnification ranging from 20 to 800,000 times, and an accelerating voltage of 0.5 to 30 kV.

## Limitations

None.

## Ethics Statements

No human subjects, animal experiments and data collected from social media platforms were involved in this work.

## CRediT authorship contribution statement

**Junfei Zhang:** Conceptualization, Methodology, Writing – review & editing. **Haoyang Han:** Data curation, Writing – original draft, Software. **Ling Wang:** Supervision.

## Data Availability

Pozzolanic-activity-experimental-dataset-of-calcined-coal-gangue-v1.0.2 (Original data).
